# UV-Photoconversion of Ethosuximide from a Longevity-Promoting Compound to a Potent Toxin

**DOI:** 10.1371/journal.pone.0082543

**Published:** 2013-12-10

**Authors:** Haeri Choi, Heather Schneider, Shannon Klum, Devon Chandler-Brown, Matt Kaeberlein, Lara Shamieh

**Affiliations:** 1 Department of Pathology, University of Washington, Seattle, Washington, United States of America; 2 Department of Biology, Regis University, Denver, Colorado, United States of America; NIEHS/NIH, United States of America

## Abstract

The anticonvulsant ethosuximide has been previously shown to increase life span and promote healthspan in the nematode *Caenorhabditis elegans* at millimolar concentrations. Here we report that following exposure to ultraviolet irradiation at 254 nm, ethosuximide is converted into a compound that displays toxicity toward *C. elegans*. This effect is specific for ethosuximide, as the structurally related compounds trimethadione and succinimide do not show similar toxicities following UV exposure. Killing by UV-irradiated ethosuximide is not attenuated in chemosensory mutants that are resistant to toxicity associated with high doses of non-irradiated ethosuximide. Non-irradiated ethosuximide extends life span at 15°C or 20°C, but not at 25°C, while irradiated ethosuximide shows similar toxicity at all three temperatures. Dietary restriction by bacterial deprivation does not protect against toxicity from irradiated ethosuximide, while non-irradiated ethosuximide further extends the long life spans of restricted animals. These data support the model that ethosuximide extends life span by a mechanism that is, at least partially, distinct from dietary restriction by bacterial deprivation and demonstrates an unexpected photochemical conversion of ethosuximide into a toxic compound by UV light.

## Introduction

Ethosuximide (2-ethyl-2-methylsuccinimide) is a member of the succinimide class of anti-convulsant drugs used for treatment of generalized absence (petit mal) epilepsy [1]. Ethosuximide is thought to act by inhibiting T-type Ca2+ channels in thalamic regions of the brain [2]. Multiple side effects associated with the gastrointestinal tract and central nervous system have been attributed to ethosuximide in patients [3], [4]. These include nausea, abdominal pain, diarrhea, anorexia, drowsiness, dizziness, fatigue, insomnia, headache, and psychotic behaviors [1]. Allergic dermatitis and rash are also common side effects associated with ethosuximide.

Ethosuximide was identified from a screen for drugs that extend life span in the nematode *Caenorhabditis elegans* [5]. At both 14 mM (2 mg/mL) and 28 mM (4 mg/mL), ethosuximide significantly extended the median life span of wild type (N2) animals maintained at 20°C by approximately 20% [6]. The effect on life span was more pronounced at 15°C, where 28 mM ethosuximide increased mean life span by 35%. Two additional succinimide anticonvulsants, trimethadione and 3,3-diethyl-2-pyrrolidinone were also found to increase life span. In addition to enhanced longevity, measures of healthspan were improved by treatment with ethosuximide, including a delay in age-associated declines in motility, reproduction, and pharyngeal pumping [6], [7].

Genetic experiments suggest that anticonvulsants enhance longevity by a mechanism distinct from dietary restriction (DR) and insulin-like signaling, the two best-characterized longevity pathways in *C. elegans*. The related anticonvulsant trimethadione further extended the life span of long-lived *eat-2(ad465)* animals [6], which consume less food than wild type animals due to a defect in pharyngeal pumping and are considered a genetic model of DR [8]. Ethosuximide and trimethadione both also further extended the life span of long-lived *daf-2(e1370)* animals, which have reduced insulin-like signaling, as well as *daf-16(mu86)* animals lacking the FOXO family transcription factor that mediates longevity control by the insulin-like signaling pathway [9], [10], [11].

At doses higher than those associated with increased life span, ethosuximide becomes toxic to *C. elegans*. Animals treated with around 84 mM ethosuximide are unable to develop into adults and die as larvae [12]. This toxicity can be partially suppressed by mutations that disrupt chemosensory function and lower doses of ethosuximide are unable to further extend the life span of long-lived chemosensory mutants. These observations have led to the model that ethosuximide increases life span by interfering with the function of chemosensory neurons [12].

We initially set out to further explore the interaction of ethosuximide with DR, using a food-deprivation paradigm referred to as bacterial deprivation (BD) in which adult animals are maintained in the complete absence of bacterial food [13], [14], [15]. We have previously shown that BD increases life span synergistically when combined with mutations that impair chemosensory function [16], and we wished to determine whether a similar effect would be observed with ethosuximide. To our surprise, we found that, under our standard conditions for life span experiments, ethosuximide treatment at concentrations previously reported to extend life span severely reduced survival of wild type animals. Here we report that this toxicity was caused by UV-irradiation of the ethosuximide as part of our protocol to growth arrest the bacterial food source. UV-mediated toxicity of ethosuximide occurs independently of medium components or bacterial food source and kills by a mechanism that appears to be distinct from high doses of non-irradiated ethosuximide. When ethosuximide is not irradiated, we were able to reproduce the previously reported life span extension and observed a temperature-dependence to this effect.

## Materials and Methods

### C. elegans Maintenance

*C. elegans* were maintained on nematode growth medium (NGM) plates with UV-killed *E. coli* OP50 as a food source. N2, *che-3 (p801)*, *osm-3 (p802)*, and *daf-16::GFP* worms were provided by the Caenorhabditis Genetics Center (CGC). *che-3 (am165)* worms were generously provided by Kerry Kornfeld.

### Reagent Preparation

Two days before the start of an assay, ethosuximide (Catalog number E7138-100G; Sigma-Aldrich, St. Louis, MO, USA) was dissolved in ddH_2_O at a stock concentration of either 1M or 2M (for life spans or resistance assays, respectively), then filter-sterilized or used to make UV-treated ethosuximide. To make UV-treated ethosuximide, the ethosuximide solution was irradiated in an uncovered petri plate for 60 minutes using a Stratalinker at 254nm then filter-sterilized. Untreated and UV-treated ethosuximide solutions were kept in foil-wrapped tubes to minimize further photoconversion. The day before the start of an assay, untreated and UV-treated ethosuximide solutions were diluted to their final concentrations. After UV-irradiation of NGM plates to kill the OP50, an equivalent volume of untreated ethosuximide, UV-treated ethosuximide, or ddH_2_O (control) was added to the surface of each NGM plate. All experimental plates were kept in foil to minimize exposure of ethosuximide to light.

### *C. elegans* Life spans

Life span assays were performed essentially as previously described [17]. Synchronized egg laying was done on nematode growth medium (NGM) plates with *E. coli* OP50 as a food source. Plates were kept at the same temperature from egg through adulthood (15°C, 20°C, or 25°C). L4 larvae were transferred to NGM containing 100 µg/mL ampicillin to prevent bacterial contamination and 50 µM 5-fluorodeoxyuridine (FUDR) to prevent egg hatching, as well as ethosuximide (0.01M or 0.03M), UV-treated ethosuximide (0.01M or 0.03M), or ddH_2_O (control). Worms were scored as alive or dead every 2-3 days. All of the life span experiments were replicated at least 6 times. Statistics are provided in **Table 1**.

**Table 1:** Statistics for life span data presented in this study.

Conc. Etho	Temp/Diet	N	Median life span (% change)	p-value	Rep.
Control	15°C	262	21		12
10 mM	15°C	310	26 (24)	0.0513	12
30 mM	15°C	236	27 (29)	*0.0173	12
10 mM (UV)	15°C	309	9 (−57)	*0.0255	12
30 mM (UV)	15°C	305	9 (−57)	*0.0187	12
Control	20°C	198	19		9
10 mM	20°C	208	21 (11)	0.0578	9
30 mM	20°C	216	21 (11)	*0.0452	9
10 mM (UV)	20°C	228	8 (−58)	*0.0089	9
30 mM (UV)	20°C	204	8 (−58)	*0.0029	9
Control	25°C	198	17		9
10 mM	25°C	210	15 (−12)	0.0665	9
30 mM	25°C	213	13 (−24)	*0.0383	9
10 mM (UV)	25°C	248	5 (−71)	*0.0109	9
30 mM (UV)	25°C	333	2 (−88)	*0.0109	9
Control	15°C	258	29		6
Control	BD/15°C	233	60 (107)	*0.0321	6
10 mM	15°C	206	32 (10)	0.0691	6
10 mM	BD/15°C	156	64 (121)	*0.0055	6
30 mM	15°C	233	34 (18)	*0.0151	6
30 mM	BD/15°C	164	64 (121)	*0.0352	6
10 mM (UV)	15°C	227	8 (−72)	*0.0218	6
10 mM (UV)	BD/15°C	172	8 (−72)	*0.0086	7
30 mM (UV)	15°C	260	8 (−72)	*0.0046	6
30 mM (UV)	BD/15°C	102	8 (−72)	*0.0038	6

The notation (UV) indicates that the ethosuximide was exposed to UV light and BD indicates that the animals were maintained in the absence of bacterial food during adulthood until death. Conc., concentration; Etho, Ethosuximide; N, number of worms in the experiment; BD, bacterial deprivation by removal of the bacterial food source during adulthood.

*p<0.05. p-values compare the experimental group with the control group of the upper row. Significant vales

### *C. elegans* Resistance Measurements

To measure resistance to ethosuximide, we adapted a protocol described by Collins, *et al* [12]. Synchronized egg laying was done on NGM plates containing ethosuximide (0.01M, 0.03M, or 0.085M), UV-treated ethosuximide (0.01M, 0.03M, or 0.085M), or ddH_2_O (control). Plates were kept at 20°C and scored for survival 5 days after the end of the synchronized egg laying period. “Proportion resistant” was defined as the number of adults divided by the total number of worms.

## Results

### UV-Irradiated Ethosuximide is Toxic to Nematodes

**Figure 1:**
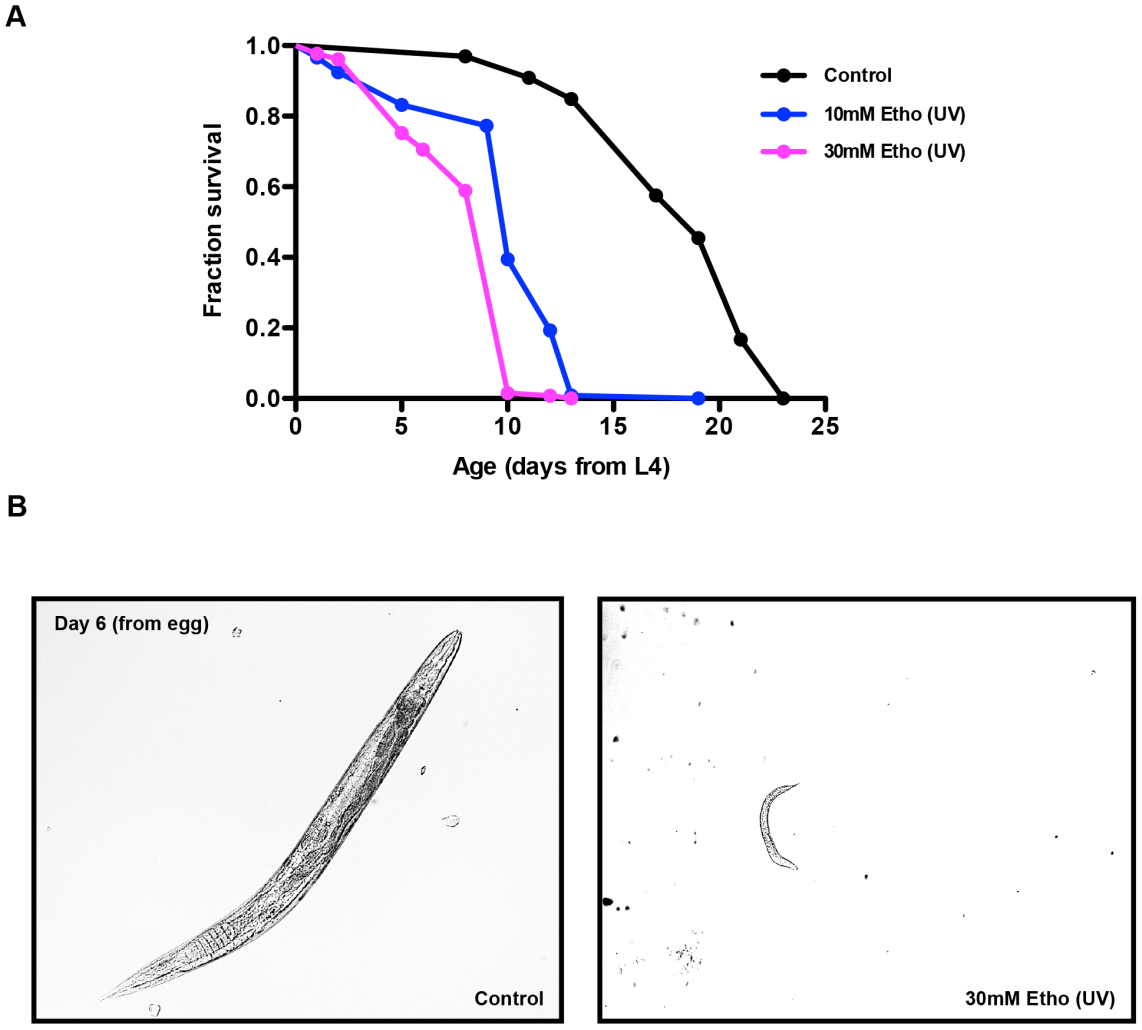
Ethosuximide treatment decreases the adult life span in *C. elegans* when UV-irradiated with the bacterial food source and NGM. (**A**) The ethosuximide (UV) treated N2 worms showed shortened life span as compared to untreated worms. (**B**) Wild-type and ethosuximide (UV) treated animals after 6 days from egg. Ethosuximide treated worms have severe defects and reduced viability. Several animals were selected, fixed onto a slide, and photographed. Images representative of the majority of animals were selected.

In order to begin to study the relationship between life span extension from ethosuximide and BD in *C. elegans*, we performed life span analyses of wild type (N2) animals treated with either 0 mM (control), 10 mM, or 30 mM ethosuximide in the nematode growth medium (NGM). Based on prior studies [6], [12], we expected to observe a significant life span extension in the ethosuximide treated animals. Surprisingly, both ethosuximide-treated cohorts displayed a significant reduction in life span accompanied by severe morphological defects (**Figure 1A, B**). Since ethosuximide treatment was initiated at the 4^th^ and final larval stage (L4), the observed toxicity could not be attributed to developmental defects occurring prior to L4.

Since our observations differed from prior reports [6], [12], we considered the possibility that variation in culture conditions could account for the different effects of ethosuximide treatment. Our standard life span protocol involves maintaining animals on NGM supplemented with UV-arrested *E. coli* OP50 and ampicillin [17]. We generally use arrested bacterial food because live *E. coli* are able to colonize the gut of the worm during aging and reduce life span [18], [19]. The UV-treatment in our protocol is accomplished by placing the NGM plate seeded with live OP50 in a Stratalinker 2400 (Stratagene Inc., La Jolla, CA) and irradiating the plates about 10 min. Evason et al. [6] reported that ethosuximide increases the life span of *C. elegans* maintained on UV-arrested OP50 as well as actively dividing OP50; however, careful examination of the methods used in that study revealed that the bacteria were UV-treated prior to being placed on the experimental plates containing ethosuximide. Thus, one difference between protocols is that in our experiments the ethosuximide is exposed to UV-irradiation while in Evason et al. [6] it was not.

**Figure 2:**
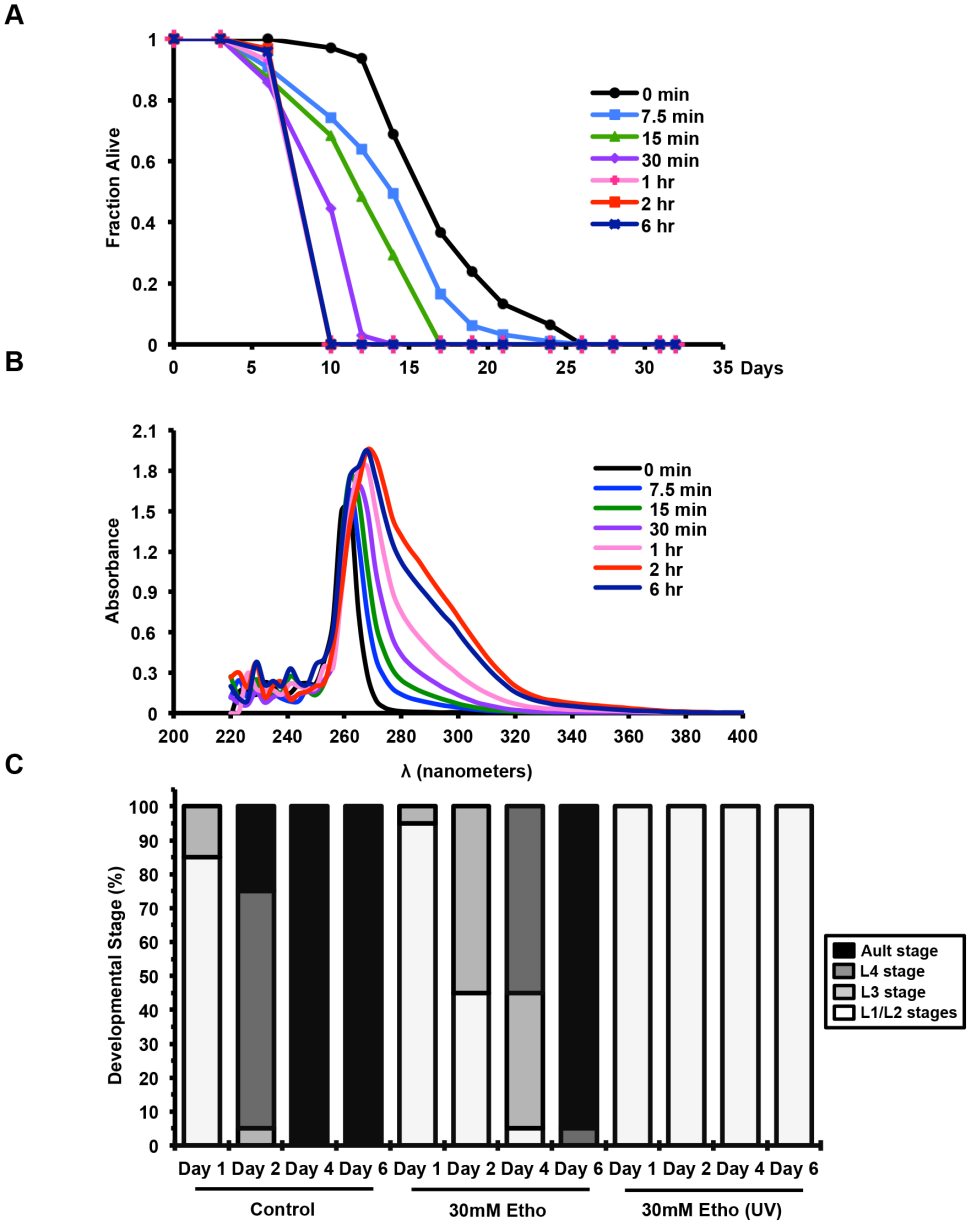
Duration of UV treatment of ethosuximide affects toxicity, absorption spectrum, and longevity of *C. elegans*. (**A**) Ethosuximide was exposed to UV-light (254nm) for varying lengths of time. L4 worms were transferred to Amp/FUDR plates containing UV-treated ethosuximide added topically to a final concentration of 10 mM and were scored for life span. Increased length of UV-exposure, led to an increased toxicity, as measured by a shortened life span. (**B**) Ethosuximide was exposed to UV light for various lengths of time (shown in graph legend). An absorption spectrum over the ranges of 220nm to 400nm was collected for the untreated and UV-treated ethosuximide solutions. (**C**) Ethosuximide delays development in wild-type (N2) worms. Eggs were transferred to NGM plates with topical addition of water (control), 30 mM ethosuximide, or 30 mM UV-irradiated ethosuximide at 20°C. The developmental stage of each worm was determined after 1, 2, 4 and 6 days as L1/L2, L3 and L4 larvae and young adult/egg-laying adult nematodes. Data from two experiments (3 independent plates each experiment) were pooled.

In order to determine whether UV-irradiation of ethosuximide causes toxicity and to separate potential interactions between ethosuximide and components of the NGM media or the bacterial food, a 1M solution of ethosuximide in water was prepared and subjected to varying amounts of UV-irradiation (see **Methods**). When applied to the surface of the NGM agar at a final concentration of 30 mM, a reduction in survival was observed that correlated with amount of irradiation (**Figure 2A**). Maximal toxicity was observed after 1 hour of irradiation in the Stratalinker, and this level of irradiation was used for all subsequent experiments. A corresponding shift in the absorption spectrum of the irradiated ethosuximide solution was also detected, consistent with the photoconversion of ethosuximide to a new product (**Figure 2B**). When eggs were allowed to hatch on NGM supplemented with ethosuximide, growth arrest prior to adulthood (**Figure 2C**) was observed, indicating that *C. elegans* larvae were also susceptible to the toxicity from UV-treated ethosuximide.

**Figure 3:**
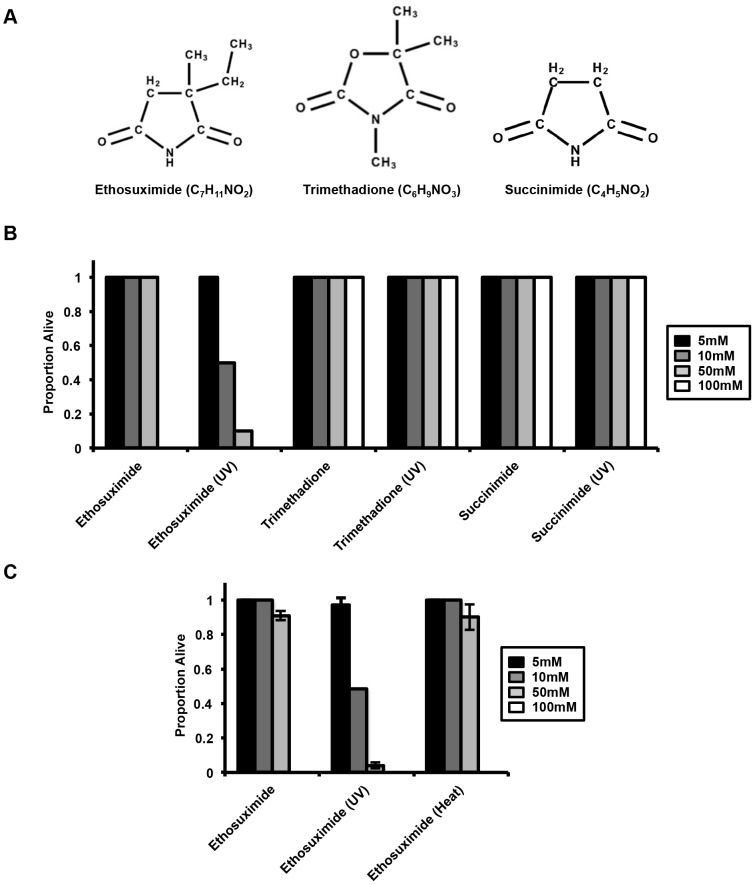
Other anticonvulsants do not exhibit similar UV-mediated toxicity in *C. elegans*. (**A**) Structures of ethosuximide, trimethadione and succinimide. (**B**) Unlike ethosuximide, UV-irradiated trimethadione and UV-irradiate succinimide caused no detectable toxicity. (**C**) Heat-treated ethosuximide also did not cause toxicity similar to UV-treated ethosuximide toxicity. L4 worms were transferred to plates containing 5, 10, 50 or 100 mM heat-treated ethosuximide, 5, 10, 50 or 100 mM UV-treated ethosuximide, or ethosuximide (control). Plates were scored for the proportion of viable worms at day 7 of adulthood. The error bars represent variation of three independent experiments (mean ± SEM).

Ethosuximide is structurally related to trimethadione, another succinimide anticonvulsant reported to extend *C. elegans* life span [6] (**Figure 3A**). In order to determine whether UV-induced toxicity of ethosuximide is a general feature of succinimide class anticonvulsants, we tested the effect of non-irradiated and irradiated trimethadione on *C. elegans.* Unlike the case for ethosuximide, UV-irradiated trimethadione exhibited no detectable toxicity (**Figure 3B**). Treating *C. elegans* with UV-irradiated succinimide also had no effect on survival. Heat treatment of ethosuximide at 95°C for 5mins also failed to induce toxicity (**Figure 3C**), suggesting that the effects result from photoconversion and are not caused by thermal breakdown of ethosuximide during irradiation.

**Figure 4:**
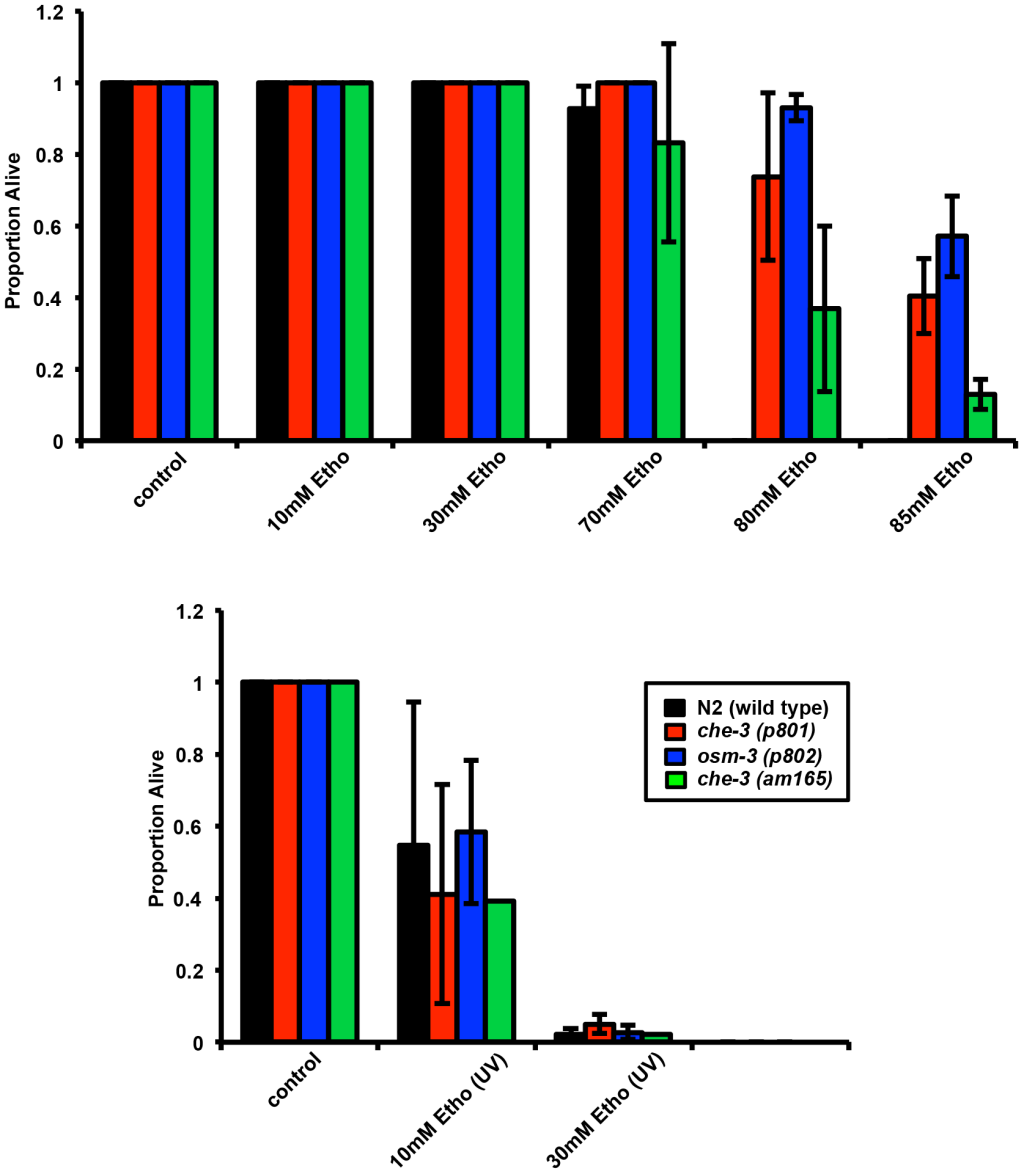
Chemosensory mutants resistant to high-dose ethosuximide do not show similar resistance to UV-treated ethosuximide. Eggs from N2, *osm-3(p802)*, *che-3(p801)*, and *che-3(am165)* worms were laid on plates containing topically added ethosuximide or UV-ethosuximide at 10, 30, 70, 80 or 85 mM, as indicated (volume matched water, control). Plates were scored for survival 5 days later.

Higher doses of non-irradiated ethosuximide (∼ 80 mM) cause reduced life span and delayed development in *C. elegans*, and mutants defective for chemosensory function show enhanced resistance to this toxicity [12]. We therefore asked whether such mutants would also be resistant to UV-irradiated ethosuximide. In contrast to their enhanced resistance to non-irradiated ethosuximide, *che-3(p801)*, *osm-3(p802)*, and *che-3(am165)* strains were not resistant to killing induced by UV-irradiated ethosuximide (**Figure 4**).

**Figure 5:**
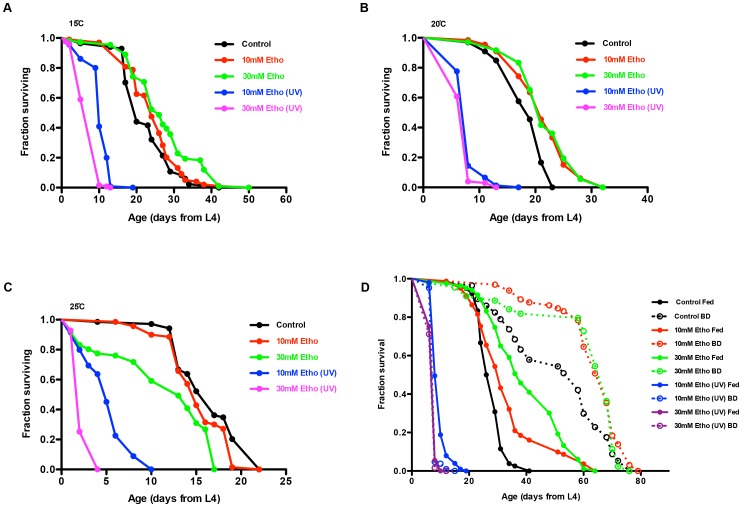
Ethosuximide treatment without UV-exposure extends the adult life span in *C. elegans* in a temperature-dependent manner. NGM containing 10-type (N2) animals at (**A**) 15°C and (**B**) 20°C, but not at (**C**) 25°C. Irradiated ethosuximide showed similar toxicity at all three temperatures. (**D**) Dietary restriction further extended the life span of ethosuximide-treated worms, but failed to rescue the short life span of UV-ethosuximide treated worms.

Having established that UV-irradiation of ethosuximide induces toxicity, we explored the effects of irradiated and non-irradiated ethosuximide on *C. elegans* as a function of temperature and food availability. We observed a temperature-dependent effect of non-irradiated ethosuximide on life span, with a significant increase in life span observed at 15°C or 20°C, and a reduction in life span from the same doses of ethosuximide at 25°C (**Figure 5A-C**). UV-irradiated ethosuximide showed similar toxicity at all three temperatures. When combined with BD at 15°C, a further increase in life span was detected from non-irradiated ethosuximide, while UV-irradiated ethosuximide resulted in comparable toxicity in BD animals compared to controls (**Figure 5D**).

## Discussion

Ethosuximide is a clinically used anticonvulsant that has received attention due to its ability to extend life span in *C. elegans*. The data presented here indicate that exposure of ethosuximide to UV light results in a photochemical reaction that produces a nematode toxin. Exposure of animals to UV-irradiated ethosuximide during adulthood dramatically reduces life span, and exposure during development arrests growth and induces death. This toxicity is at least somewhat specific for ethosuximide, as the related compounds trimethadione and succinimide do not show similar toxicity following UV-irradiation. The mechanism of killing from UV-irradiated ethosuximide appears to be distinct from toxicity induced by high doses of non-irradiated ethosuximide and occurs independently of the bacterial food source. In contrast to unirradiated ethosuximide, UV-treated ethosuximide failed to extend life span at any of the doses tested. It is possible, however, that a much lower dose of UV-treated ethosuximide might extend life span, either through a hormesis-like mechanism or by inducing the same mechanism of life span extension as unirradiated UV-ethosuximide. Additional studies will be required to assess this possibility.

Although it was not our initial intention to study UV-mediated conversion of ethosuximide into a toxic compound, these observations reinforce the importance of rigorously controlling for experimental conditions during longevity studies of *C. elegans*. It is now widely recognized that the use of dividing *E. coli* as a food source can impact the outcome of life span experiments. For this reason, we have chosen to utilize growth-arrested *E. coli* whenever feasible, in order to avoid studying interventions that extend life span solely due to antibiotic effects on the food source. As this study points out, however, even the methods used to arrest bacterial growth (UV irradiation in this case) can also impact the outcome of the experiment.

Another important variable in *C. elegans* longevity studies is temperature. Recent reports have indicated that the effects of both genetic and pharmacological interventions can have opposite effects on life span at 15°C and 25°C [20], [21], [22], [23]. As in these other cases, non-irradiated ethosuximide has a temperature-dependent effect on life span. Life span is significantly extended by non-irradiated ethosuximide at 15°C but is shortened by the same concentration of ethosuximide at 25°C. This is identical to the temperature-dependent effect of caffeine on life span [21], perhaps suggesting that caffeine and ethosuximide act on longevity through similar mechanisms.

Our initial goal for this study was to determine the interaction between dietary restriction by BD and ethosuximide. Based on the prior report that trimethadione extends life span when combined mutation of *eat-2* [16], we predicted that ethosuximide would also extend life span when combined with dietary restriction. Consistent with this prediction, ethosuximide further extended the long life span of BD animals, supporting the model that dietary restriction and anticonvulsants act through distinct mechanisms to promote longevity in *C. elegans*. BD animals were not resistant to the killing by UV-irradiated ethosuximide, however.

The relevance of these findings for human patients taking ethosuximide is unclear. It is unlikely that ethosuximide used in a clinical setting would be exposed to levels of UV light comparable to those utilized in this study; however, it is not unreasonable to expect that low levels of UV exposure due to sunlight or other environmental sources of radiation could occur either before or after the drug is taken by a patient. Interestingly, one common side effect of ethosuximide is a skin rash on the face and nose that is associated with exposure to sunlight. Thus, it may be that low level photoconversion of ethosuximide from sunlight contributes to skin irritation in people taking this drug.

## Conclusions

We were able to successfully reproduce the previously reported life span extension from the anticonvulsant ethosuximide in *C. elegans*. Ethosuximide extends life span at 15°C, but the same dose shortens life span at 25°C. This life span extension is additive with dietary restriction by BD. When exposed to UV light, ethosuximide is converted into a toxic compound that dramatically shortens life span.
